# Residual Force Enhancement Is Present in Consecutive Post-Stretch Isometric Contractions of the Hamstrings during a Training Simulation

**DOI:** 10.3390/ijerph18031154

**Published:** 2021-01-28

**Authors:** Neil D. Chapman, John W. Whitting, Suzanne Broadbent, Zachary J. Crowley-McHattan, Rudi Meir

**Affiliations:** 1 School of Health and Human Sciences, Southern Cross University, Lismore, NSW 2480, Australia; John.whitting@scu.edu.au (J.W.W.); sbroadbe@usc.edu.au (S.B.); zac.crowley@scu.edu.au (Z.J.C.-M.); rudi.meir@scu.edu.au (R.M.); 2 Faculty of Health Sciences and Medicine, Bond University, Robina, QLD 4229, Australia; 3 School of Health and Behavioural Sciences, University of the Sunshine Coast, Sippy Downs, QLD 4556, Australia

**Keywords:** residual force enhancement, hamstrings, training simulation, electromyography, history dependence, muscle, in vivo

## Abstract

Residual force enhancement (rFE) is observed when isometric force following an active stretch is elevated compared to an isometric contraction at corresponding muscle lengths. Acute rFE has been confirmed in vivo in upper and lower limb muscles. However, it is uncertain whether rFE persists using multiple, consecutive contractions as per a training simulation. Using the knee flexors, 10 recreationally active participants (seven males, three females; age 31.00 years ± 8.43 years) performed baseline isometric contractions at 150° knee flexion (180° representing terminal knee extension) of 50% maximal voluntary activation of semitendinosus. Participants performed post-stretch isometric (PS-ISO) contractions (three sets of 10 repetitions) starting at 90° knee extension with a joint rotation of 60° at 60°·s^−1^ at 50% maximal voluntary activation of semitendinosus. Baseline isometric torque and muscle activation were compared to PS-ISO torque and muscle activation across all 30 repetitions. Significant rFE was noted in all repetitions (37.8–77.74%), with no difference in torque between repetitions or sets. There was no difference in activation of semitendinosus or biceps femoris long-head between baseline and PS-ISO contractions in all repetitions (ST; baseline ISO = 0.095–1.000 ± 0.036–0.039 Mv, PS-ISO = 0.094–0.098 ± 0.033–0.038 and BFlh; baseline ISO = 0.068–0.075 ± 0.031–0.038 Mv). This is the first investigation to observe rFE during multiple, consecutive submaximal PS-ISO contractions. PS-ISO contractions have the potential to be used as a training stimulus.

## 1. Introduction

There is much interest in the prevention and rehabilitation of hamstring strain injuries. Hamstring strain injuries have a high incidence, particularly during high-speed running [1]. The function of the hamstring muscles during high-speed running is vigorously debated. One theory proposes that in high-speed running, the hamstring muscles act eccentrically during the late swing phase of the gait cycle [2]. In contrast, the alternate postulation states that the hamstrings remain predominantly isometric during the late swing phase and act isometrically during foot contact [2]. Much of our current understanding of the dynamic function of the hamstrings is based on kinematic and kinetic investigations that have measured the changes in distance between osteotendinous attachments, sometimes with the calculation of corresponding joint moments [3,4,5,6,7,8,9]. Methodological limitations exist wherever inferences about the behaviour of the contractile element are based on the change in distance between the musculotendinous origin and insertion [2]. These inferences do not account for the behaviour of the series elastic element and other non-contractile tissues such as aponeuroses and fascial tissues [2]. This ongoing debate concerning hamstring function, although worthwhile, may prove to be somewhat academic if the dynamic functioning of the hamstrings during the gait cycle of high-speed running is found to be specific to the individual [10].

Uncertainty over the dynamic function of the hamstrings has led to conjecture over appropriate training methods for the hamstring muscles, be it for performance, injury prevention or rehabilitation. Eccentric strength, fascicle length and neuromuscular functioning have been identified as crucial modifiable risk factors for injury [11], and are often the focus of injury prevention programs. Flywheel training [12] and the Nordic hamstring exercise [13] are examples of eccentrically biased training methods purported to be effective in injury risk minimization [14,15]. However, if isometric actions occur at critical end-range moments during dynamic tasks, then there is cause to incorporate isometric specific exercise [16]. Van Hooren and Bosch [17] suggest that isometric exercises such as the Roman chair hold (and variations) can generate sufficient overload, while maintaining complimentary transfer for improvements in performance and reduction in injury risk. Van Hooren and Bosch hypothesized that high-intensity isometric contractions may prove more effective than eccentric contractions in preventing hamstring strain injury during high speed running [2,18]. However, Van Hooren and Bosch advocated for the use of both eccentric and isometric contractions to be included in hamstring strain injury prevention programs [2,17,18]. Therefore, the use of both eccentric and isometric contractions has the potential to be most beneficial for reducing hamstring injury risk.

The post-stretch isometric (PS-ISO) contraction, which combines both eccentric and isometric stimuli, may provide the benefits of both contraction modes. A PS-ISO contraction is initiated with an isometric contraction at a shorter musculotendinous unit length, then moved through an active stretch phase, ending with a sustained isometric contraction at the new longer muscle length [19]. Torque output peaks during the active stretch phase before normalizing somewhat during the final sustained isometric steady-state. Due to history-dependent effects, the torque observed during the PS-ISO steady-state is consistently greater than isometric torque without active stretch [19]. This elevated PS-ISO torque is referred to as residual force enhancement (rFE) [20], which has been observed in vitro in single-fibre and whole-muscle preparations [20,21,22,23,24,25], and in vivo with electrical stimulation [26,27,28,29] and voluntary contractions [27,28,30,31,32,33]. The magnitude of rFE is greater at joint angles indicative of longest muscle lengths [23,34,35], and increases with increasing stretch magnitudes [36,37,38].

rFE has been observed in the hamstring muscle group in maximal and submaximal voluntary PS-ISO contractions [30]. The maximal PS-ISO steady-state torque was found to be almost 9% greater than the baseline isometric torque without prior stretching. A 39% increase in torque between isometric steady-state and PS-ISO torque was found using a submaximal PS-ISO contraction intensity (50% activation). Ultrasonographic confirmation of contractile element lengthening (eccentric contraction), coupled with a lack of increased muscle activation, led to the postulation that mechanical history-dependent effects from the giant protein titin increased stiffness during the active stretch of the PS-ISO contractions. The authors reason that the resultant torque increase via titin contribution is congruent with the titin elasticity theory [38], which states that the giant muscle protein titin is activated and increases stiffness within the muscle during muscle stretch. The enhanced force resulting from increased titin stiffness is maintained during the steady-state isometric contraction following muscle stretch (eccentric contraction) [38]. Furthermore, the enhanced force contributed via titin is present without increased muscle activation [38]. Thus, PS-ISO contractions, which involve both eccentric and isometric contraction modes, have the potential to significantly increase isometric torque output without increased muscle activation of the hamstring muscle group.

It has been shown that chronic use of eccentric contractions results in increased eccentric strength and increased fascicle length [39], often concomitantly with a rightward shift in the optimal operating angle on the length–tension curve [40]. The chronic use of isometric exercise at long musculotendinous unit lengths has been shown to increase isometric strength, pennation angle and fascicle length, and cause hypertrophy [41]. A broadening of the plateau region of the length–tension curve has also been observed in chronic isometric training at long musculotendinous unit lengths [42]. The potential benefits of combining eccentric and isometric contraction modes using PS-ISO contractions in chronic resistance training is unknown. However, it is reasonable to hypothesize that such a chronic stimulus may provide some or all of the benefits derived individually from chronic eccentric and isometric stimuli alone.

Notwithstanding this, before investigating the chronic training effects of PS-ISO contractions, it is critical to understand the acute effects of PS-ISO contractions experienced as a training stimulus. Presently, it is unknown whether rFE endures beyond single PS-ISO contractions. Prior to a training study being undertaken, it is necessary to investigate whether rFE persists in the hamstring muscle group during a series of consecutive PS-ISO contractions across multiple sets, as applied in a training simulation. Therefore, this study aimed to observe the presence of rFE during multiple sets of a series of consecutive submaximal PS-ISO contractions using the hamstring muscle group. It was hypothesized that rFE would be observed across all repetitions of activation-matched contractions performed in series as a training simulation.

## 2. Materials and Methods

Prior to recruitment, an a priori calculation was undertaken that calculated *n* = 10. Ten recreationally trained [43] participants (seven males, three females; age 31.00 years ± 8.43 years) provided written informed consent to participate in the study. Participants were classified as novice for performing PS-ISO contractions at the time of data collection. All participants were confirmed to be free from diagnosed lower-limb musculoskeletal injury and neurologic conditions in the preceding 12 weeks. The study was approved by the Institutional Human Research Ethics Committee (ECN: 2019/090).

Each participant assumed a prone position on a Biodex System 3 isokinetic dynamometer (Biodex Medical Systems, Shirley, NY, USA), which recorded torque measurements for all experiments. The participant’s hip angle was confirmed to be between 170° and 180° (180° represents neutral hip position) via goniometer (J.A. Preston Corporation, Clifton, NJ, USA). The goniometer was centred on the greater trochanter of the involved hip and aligned with the lateral midline of the abdomen and lateral midline of the femur. The axis of rotation of the participant’s involved knee was aligned with the axis of rotation of the dynamometer. The ankle cuff was attached 25 mm above the dorsal surface of the foot. Inelastic straps were placed over the L4/5 area to mitigate extraneous movements during contractions.

Surface electromyography (sEMG) signals of the semitendinosus (ST) and biceps femoris long-head (BF*lh*) muscles were recorded during all trials using a Trigno Wireless sEMG system with double differentiated surface electrodes (Delsys, Natick, MA, USA). The electrodes were placed as per the SENIAM guidelines [44]. The ST electrode was placed on the muscle at 50% of the distance along the line between the ischial tuberosity and the medial epicondyle of the tibia. The BF*lh* electrode was placed on the muscle at 50% of the distance along the line between the ischial tuberosity and the lateral epicondyle of the tibia. The electrode locations were prepared by first shaving and abrading, then wiping the site with alcohol wipes. In addition to double-sided electrode-skin interface adhesives, surgical adhesive tape was used to secure the electrodes to the skin.

The Biodex data was sampled at 1000 Hz using a 12-bit analogue-to-digital converter (PowerLab System 16/35, ADInstruments, Bella Vista, Australia). The sEMG signals were sampled at 2000 Hz (bandpass filtered at 10–500 Hz). The Biodex and sEMG were synchronized with LabChart software (Pro Modules 2014, version 8, ADInstruments, Bella Vista, Australia).

### 2.1. Protocols and Measurements

**Figure 1:**
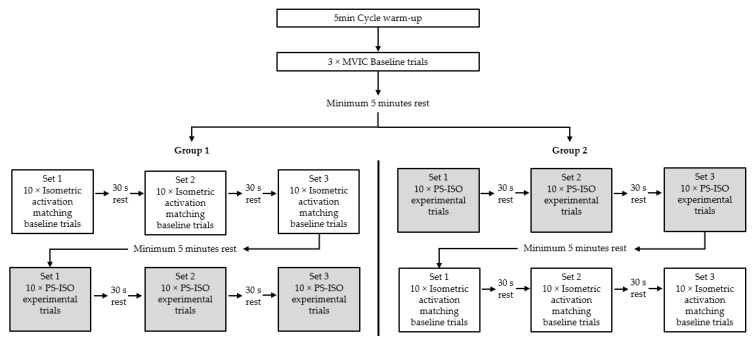
Baseline and experimental protocols for Group 1 and Group 2. Participants were randomly allocated into each group.

The experimental protocol is visualized in Figure 1. A 5 min generalized warm-up using a cycle ergometer was completed. No static or dynamic warm-up stretching was completed as part of the warm-up protocol. Following the generalized warm-up, each participant performed three baseline maximal voluntary isometric contractions (MVIC) of the knee flexors (5 s duration) at 150° knee flexion (180° being representative of terminal knee extension). To ensure that MVIC attempts were maximal, each participant was provided with verbal encouragement and visual feedback of the torque traces on a computer monitor within a direct line of sight of the participant. The activation-matching intensity, 50% ± 5% MVIA, was calculated using the activation of the ST, as the ST has been shown to have the greatest activation compared with other hamstring muscles during eccentric contractions [45]. The baseline MVIC mean of ST root mean square (RMS) amplitude (mV) sEMG (sEMG_RMS_: moving average window = 50 ms) was derived from a 3 s epoch, corresponding with a 2–4 s window in the MVIC baseline contractions. The values of 50% ± 5% were entered into the LabChart software to be visualized as guidelines on a computer monitor located in front of the participant. Each participant then performed three sets of 10 baseline activation matching isometric contractions of the knee flexors (7 s duration at 150° knee flexion), for a total of 30 contractions [46].

The experimental condition consisted of three sets of 10 repetitions of activation-matched PS-ISO contractions, for a total of 30 PS-ISO contractions. Each activation-matching PS-ISO repetition was initiated isometrically at 90° of knee flexion (using the verbal trigger of “pull”), followed by an active stretch over a joint excursion of 60° at a constant angular velocity of 60°·s^−1^. This was then immediately followed by an activation-matched post-stretch isometric contraction at 150° of knee flexion of 7 s duration (using the verbal trigger of “match”). After each repetition, the dynamometer arm automatically and immediately returned to the starting position with no effort required by the participant (using the verbal trigger “relax”).

To ensure that all activation-matching attempts (PS-ISO and baseline) were within ± 5%, each participant was provided with verbal encouragement and real-time visual feedback of the ST sEMG_RMS_ trace on a computer monitor located within a direct line of sight of the participant. Participants rested for 3 s between activation matching repetitions, 30 s between activation matching sets and a minimum of 5 min between the isometric baseline and the PS-ISO experimental condition. A counterbalanced design was used whereby 50% of participants completed the baseline isometric activation-matched contractions prior to experimental PS-ISO activation-matched contractions. The remaining 50% of participants completed the experimental PS-ISO activation-matched contractions before the baseline isometric activation-matched contractions.

### 2.2. Data Analysis

The mean torque output (Nm) was derived from a 3 s epoch corresponding to 3–5 s for each baseline isometric repetition and 3–5 s for each PS-ISO steady-state repetition. The mean sEMG_RMS_ (mV) was derived from a 3 s epoch corresponding to 3–5 s for each experimental trial. The rFE magnitude was defined as the absolute torque increase (Nm) and as a percentage change from the activation-matched isometric baseline contraction at 150° knee flexion. The following equation, previously used by Dalton et al. [47], was used to calculate the percentage change for rFE:

$$\mathrm{rFE}\%\Delta \;=\;\left[\frac{\left(\;\mathrm{isometric}\;\mathrm{torque}\;\mathrm{Nm}\;\mathrm{following}\;\mathrm{active}\;\mathrm{lengthening}-\mathrm{baseline}\;\mathrm{isometric}\;\mathrm{torque}\;\mathrm{Nm}\right)}{\mathrm{baseline}\;\mathrm{isometric}\;\mathrm{torque}\;\mathrm{Nm}}\right]\;\times \;100\%$$

### 2.3. Statistical Analysis

All variables of interest were tested using the Shapiro–Wilk tests and found to be normally distributed. A 2 × 3 × 10 repeated-measures ANOVA was used to assess the difference between condition (2), sets (3) and repetitions (10) for torque and sEMG_RMS_ of ST and BF*lh*. Where a main effect or interaction was found, a post-hoc test with Bonferroni corrections was conducted to further determine where the differences existed between conditions, set and repetitions. These calculations were made for all repetitions within a set, and all sets of repetitions. Effect sizes were calculated using partial *η*^2^ (0.30 = small, 0.50 = medium, >0.50 = large effect size) [48]. Significance was determined based on an α = 0.05. Descriptive data in figures are reported as mean values.

## 3. Results

### 3.1. Torque

**Figure 2:**
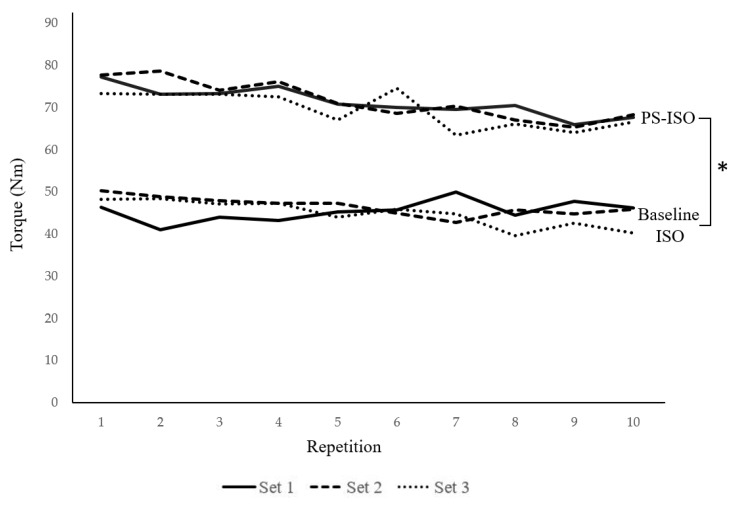
Mean baseline isometric and PS-ISO torque during 10 consecutive repetitions over three sets. * indicates a significant increase in mean torque from baseline isometric contractions to PS-ISO contractions across all repetitions and all sets (*p* ≤ 0.001).

The mean torque (Nm) for each repetition is presented in Table 1. A main effect of the contraction type revealed that the activation-matched PS-ISO contraction torque was significantly greater than the activation-matched baseline isometric torque (baseline ISO; CV = 36.67–69.50; PS-ISO; CV = 20.27–38.61, F = 32.558, *p* = <0.001, partial $\eta^{2}$ = 0.783). However, no significant main effect of set (F = 0.640, *p* = 1.000, partial $\eta^{2}$ = 0.138) or repetition (F = 3.555, *p* = 1.00, partial $\eta^{2}$ = 0.970) was found. All interactions between contractions, sets and repetitions were non-significant (F = 1.197–3.442, *p* = 0.060–1.000, partial $\eta^{2}$ = 0.340–0.989). To examine the main effect of contraction, the post-hoc analysis revealed that there were no differences in baseline isometric torque output between repetitions (F = 3.422, *p* = 1.00, partial $\eta^{2}$ = 0.969) or sets (F = 2.058, *p* = 1.000, partial $\eta^{2}$ = 0.340). Further, there were no differences in torque output in PS-ISO between repetitions (F = 0.697, *p* = 1.000, partial $\eta^{2}$ = 0.863) or sets (F = 0.193, *p* = 1.000, partial $\eta^{2}$ = 0.046). However, a significant difference between contraction modes at each repetition within each set was found (F = 15.474–41.735, *p* = <0.001–0.003, partial $\eta^{2}$ = 0.632–0.823). This demonstrated that the PS-ISO torque was consistently elevated above the baseline isometric torque (Figure 2).

### 3.2. sEMG_RMS_

**Figure 3:**
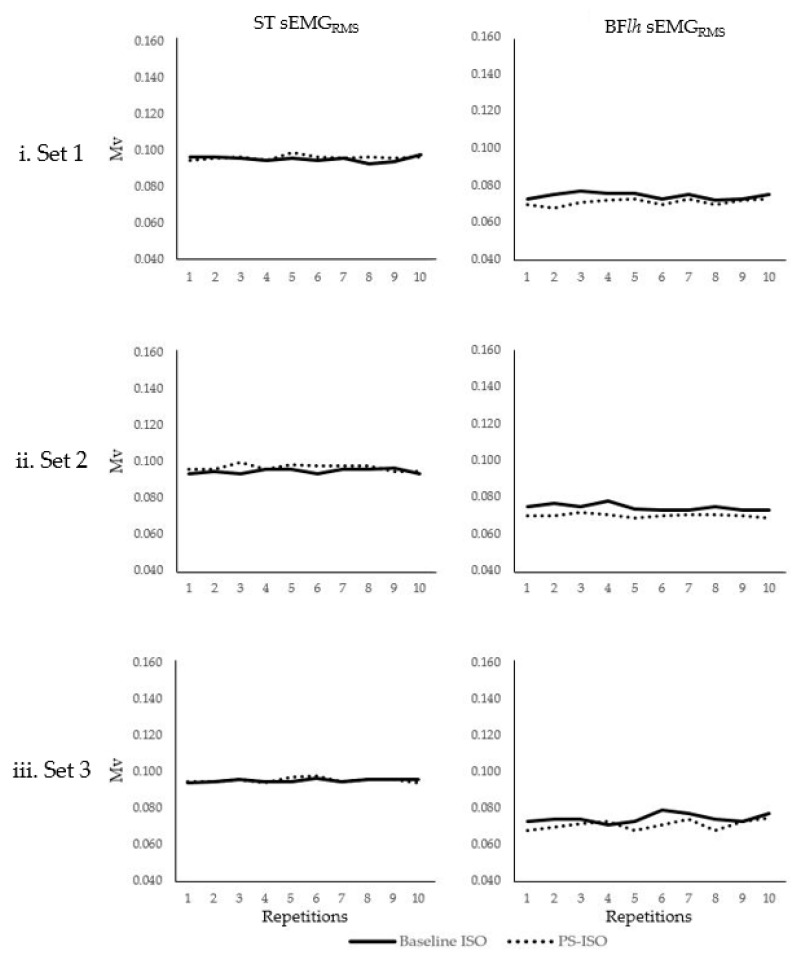
Mean baseline isometric and PS-ISO muscle activation (sEMG_RMS_) measured in millivolts (Mv) of semitendinosus (ST) and biceps femoris long-head (BF*lh*) muscles during 10 consecutive repetitions during 3 sets (**i. Set 1**, **ii Set 2** and **iii Set 3**). No difference in muscle activation was observed between baseline isometric and PS-ISO contractions for all repetitions and sets of semitendinosus (*p* = 0.590) and biceps femoris long-head (*p* = 0.198). ST sEMG_RMS_ is depicted in the left column and BF*lh* sEMG_RMS_ is depicted in the right column for Set 1 (**i Set 1**), Set 2 (**ii. Set 2**) and Set 3 (**iii. Set 3**). Repetitions in each set are visualized on the *x* axis and sEMG_RMS_ on the *y* axis of each graph.

No main effect of contraction type (ST; baseline ISO = 0.095–1.000 ± 0.036–0.039 Mv, PS-ISO = 0.094–0.098 ± 0.033–0.038, F = 0.312, *p* = 0.590, partial $\eta^{2}$ = 0.033 and BFlh; baseline ISO = 0.068–0.075 ± 0.031–0.038 Mv, PS-ISO = 0.071–0.079 ± 0.030–0.038, F = 1.931, *p* = 0.198, partial $\eta^{2}$ = 0.177), set (ST; F = 1.280, *p* = 1.000, partial $\eta^{2}$ = 0.242 and BFlh; F = 0.247, *p* = 1.000, partial $\eta^{2}$ = 0.058) or repetition (ST; F = 1.000, *p* = 1.000, partial $\eta^{2}$ = 0.900 and BFlh; F = 1.111, *p* = 1.000, partial $\eta^{2}$ = 0.909) were found for muscle-activation variables. No interactions were found for all muscle activations between main effects of contraction, sets and repetitions for ST and BFlh (ST; F = 1.000–1.500, *p* = 0.110–1.000, partial $\eta^{2}$ = 0.600–0.700 and BFlh; F = 0.224–9.472, *p* = 0.052–1.000, partial $\eta^{2}$ = 0.343–0.988) (Figure 3).

## 4. Discussion

This is the first study to confirm rFE, in the absence of increased muscle activation, in the hamstrings during multiple and consecutive submaximal PS-ISO contractions performed as a training simulation. Notably, the hypotheses were supported. Interestingly, the magnitude of rFE in the current study (55%), was greater than that of the previous investigation of the hamstring muscle group (39%) [30] and of that found in other lower-limb muscles (25%) [49]. It is evident from the findings that an increase in muscle activation cannot account for the elevated PS-ISO torque during this series of PS-ISO contractions. Furthermore, in this submaximal PS-ISO condition (50% MVIA), there was no reduction in the magnitude of rFE during multiple, consecutive PS-ISO contractions. Thus, we posit that mechanical factors influenced by history-dependent muscle contractions, such as titin stiffness, are likely primarily responsible for the torque increase in the experimental condition. These findings are the first to observe the repeatability of enhanced torque (i.e., rFE) using PS-ISO contractions as per a traditional training simulation. These findings may have practical application to chronic resistance-training exercises focused on hamstring injury prevention.

The current study demonstrates that the mechanisms responsible for rFE persist beyond a single bout. We posit that this mechanism is most likely to be the giant protein titin. Titin elasticity theory states that during muscle stretch, titin binds to the actin filament, reducing free spring length. This, in turn, increases sarcomeric stiffness, thereby contributing additional passive force to the total force output [38,50,51,52]. Furthermore, as a consequence of titin involvement, it has been suggested that forces in the enhanced state come at a reduced metabolic cost [53]. Although muscle lengthening was not directly observed in the current study, the assertion that titin contributed force during an active stretch in the current study is further supported by the following:

(i) A lack of increased muscle activation during PS-ISO contractions, which suggests that increased torque was primarily mechanical in nature and minimally influenced by neuromechanical factors [54,55,56].
(ii) The levels of isometric pre-activation in the current study were sufficient to influence muscle lengthening and activation of titin. Previous investigations have suggested that modulation of muscle lengthening is influenced by muscle–tendon interaction [57] and the elimination of muscle slack [58] during the isometric pre-activation phase. The influence of sufficient isometric pre-activation on muscle stretch and magnitude of rFE has been demonstrated in maximal and submaximal PS-ISO contractions [30,50,59].
(iii) A recent investigation of submaximal PS-ISO contractions was undertaken by the current authors, which directly confirmed muscle lengthening of BF*lh* via ultrasound during PS-ISO contractions [30]. That study used the same body position, joint excursion, angular velocity and submaximal contraction intensity as the current study [30]. We therefore surmise that it is highly likely that muscle lengthening, and therefore engagement of titin, took place in the current study. However, it is acknowledged that other non-contractile elements, such as tendons and aponeuroses, may also have contributed to the enhanced PS-ISO steady-state force.

This study demonstrates that the phenomenon responsible for rFE is reproducible during consecutive PS-ISO contractions, similar to standard resistance-training protocols [60]. The potential benefits of a contraction mode that incorporates an eccentric stimulus, resulting in an enhanced isometric steady-state force at a reduced metabolic cost [53], are intriguing. Evidence suggests that with chronic use of eccentric contractions, increased eccentric strength and increased fascicle length [39] often occur concomitantly with a rightward shift in the optimal operating angle on the length–tension curve [40]. These changes to the structure and behaviour of the muscle have been found to increase the resilience of the hamstring to strain injury [61]. However, athlete compliance with eccentrically biased programs remains an issue [62]. Notwithstanding the evidence for the benefits of eccentric contractions in hamstring resilience, the importance of isometric hamstring strength has recently been proposed [2,17,18,63]. It is known that with chronic implementation of isometric exercise at long musculotendinous unit lengths, increases in isometric strength, pennation angle, fascicle length and hypertrophy occur [41]. Furthermore, a broadening of the plateau region of the length–tension curve is also known to occur with the implementation of chronic isometric training at long musculotendinous unit lengths [42], which have the potential to provide similar prophylactic effects to hamstring strain injury. The results of the current study indicate that the use of post-stretch isometric contractions that incorporate both an isometric and eccentric stimulus results in enhanced force output of the muscle during both the eccentric and post-eccentric isometric steady-state phases. This increase in force was found to occur in the absence of increased muscle activation, hence, PS-ISO contractions that result in rFE may be more metabolically efficient when compared to eccentric or isometric contractions to achieve similar levels of force [53,64]. However, the effects of the combined use of these contraction modes shown to be beneficial to hamstring injury resilience are currently unknown.

Therefore, based on the findings of this study, future research should test the efficacy of the chronic use of PS-ISO contractions in hamstring strain injury prevention. The potential exists that with the chronic use of PS-ISO contractions, athletes could experience benefits including increased muscle hypertrophy, increased eccentric and isometric strength, increased fascicle length and a rightward shift or broadening of the plateau of the optimum angle in the length–tension curve [12,41,65,66,67].

An intermediate consideration, however, is the possibility that chronic performance of PS-ISO contractions in resistance training may modify the history dependence of force. For instance, a modification to the history dependence of force could result in a decrease in rFE. This is because it has been hypothesized that increased fascicle length in a muscle may result in less stretching of titin per sarcomere, leading to a reduction in passive force following active lengthening and a reduction in rFE [49]. The influence of muscle architecture changes on rFE has recently been investigated, with varied effects found [49,68,69]. For example, Hinks, Davidson, Akagi and Power [67] found no significant changes in rFE following chronic isometric training at long or short muscle lengths, despite the fascicle length increasing and decreasing, respectively [67]. In contrast, evidence of the effect of concentric and eccentric training on rFE is less certain. Chen and Power [49] observed an increase in rFE magnitude following concentric training and a decrease following eccentric training that corresponded to changes in fascicle lengths. These findings are tempered by their conclusion that their results were influenced by a change in non-responder rates and antagonist co-activation between conditions [49]. Thus, the influence of chronic isometric, concentric and eccentric training on history dependence of force deserves greater attention. Notwithstanding the need for further investigation of the influence that resistance training may have on rFE, it is clear that alternative injury-prevention strategies are needed to arrest the high levels of hamstring strain injury incidence. PS-ISO contractions have the potential to benefit from both eccentric and isometric stimulus, with the added benefits of enhanced torque output (rFE) at a lower metabolic cost. Therefore, an investigation into the effects of a training study using PS-ISO contractions of the hamstrings is recommended.

## 5. Conclusions

This is the first investigation to observe rFE during multiple, consecutive submaximal PS-ISO contractions across multiple sets. The absence of increased muscle activation during PS-ISO contractions suggests that mechanical phenomena, such as increased stiffness in the giant protein titin, were the primary contributors to increased passive force in the enhanced state [30]. Debate exists over the most effective and efficient ways to improve hamstring function and injury resistance, yet the use of chronic eccentric and isometric training is often advocated for hamstring injury prevention [17]. Eccentric and isometric strength training are known to share similar benefits (increase in strength, fascicle length increase and a broadening or rightward shift in the optimum angle of peak torque [12,41,65,66,67]). PS-ISO contractions incorporate both an eccentric and isometric stimulus, with the added benefits of enhanced isometric torque at reduced metabolic cost. It would appear intuitive that the use of PS-ISO contractions in resistance training could combine the benefits of eccentric or isometric training effectively and efficiently. Investigation of the effects of chronic resistance training that uses PS-ISO contractions, particularly in the hamstring muscles, is certainly warranted.

## Figures and Tables

**Table 1:** Torque values (Nm) for baseline and PS-ISO contractions.

	Set 1		Set 2			Set 3	
Rep	BL	PS-ISO	BL	PS-ISO	BL		PS-ISO
1	46.43 (27.00)	77.29 (21.97) *	50.29 (24.57)	77.86 (24.03) *	48.34 (20.11)		73.40 (19.08) *
2	41.18 (24.47)	73.20 (21.01) *	48.92 (22.19)	78.81 (18.50) *	48.42 (17.76)		73.29 (17.78) *
3	44.09 (27.79)	73.35 (26.20) *	48.07 (26.55)	74.22 (20.11) *	47.15 (17.77)		73.27 (19.16) *
4	43.29 (30.09)	75.12 (23.98) *	47.43 (23.02)	76.22 (22.65) *	47.43 (20.40)		72.59 (17.60) *
5	45.28 (24.11)	70.84 (27.35) *	47.45 (23.19)	71.01 (20.04) *	44.06 (18.74)		67.19 (19.34) *
6	45.81 (28.76)	70.14 (24.25) *	45.02 (21.67)	68.74 (20.98) *	46.01 (17.07)		74.62 (15.12) *
7	50.11 (30.22)	69.63 (23.06) *	42.83 (23.85)	70.43 (19.59) *	44.85 (18.98)		63.48 (17.55) *
8	44.63 (25.91)	70.53 (21.78) *	45.84 (21.14)	67.13 (18.81) *	39.74 (22.10)		66.23 (15.38) *
9	47.90 (30.78)	66.01 (20.04) *	44.91 (17.86)	65.38 (20.58) *	42.63 (18.06)		64.23 (16.61) *
10	46.28 (27.02)	67.72 (20.76) *	45.94 (20.90)	68.34 (21.43) *	40.25 (18.69)		66.66 (15.98) *

Note. Torque values mean (SD). All values in Nm. * indicates a significant difference between baseline (BL) and post-stretch isometric (PS-ISO) repetitions.

## Data Availability

Publicly available datasets were analyzed in this study. This data can be found here: https://www.researchgate.net/profile/Neil_Chapman4/research.
